# A human oral commensal-mediated protection against Sjögren’s syndrome with maintenance of T cell immune homeostasis and improved oral microbiota

**DOI:** 10.1038/s41522-025-00654-5

**Published:** 2025-01-16

**Authors:** Yu-Chao Tseng, Kai-Sheng Liao, Wei-ting Lin, Chin Li, Chia-Bin Chang, Jie-Wei Hsu, Chin-Pui Chan, Chun-Ming Chen, Hon-Pin Wang, Hsiu-Chuan Chien, Jann-Tay Wang, Song-Chou Hsieh, Shu-Fen Wu

**Affiliations:** 1 Division of Allergy, Immunology, and Rheumatology, Department of Internal Medicine, Ditmanson Medical Foundation Chia-Yi Christian Hospital, Chiayi, Taiwan; 2 Department of Medical Research, Ditmanson Medical Foundation Chia-Yi Christian Hospital, Chiayi, Taiwan; 3 Department of Biomedical Sciences and Institute of Molecular Biology, National Chung Cheng University, Chiayi, Taiwan; 4 Department of Pathology, Ditmanson Medical Foundation Chia-Yi Christian Hospital, Chiayi, Taiwan; 5 Department Oral and Maxillofacial Surgery, Ditmanson Medical Foundation Chia-Yi Christian Hospital, Chiayi, Taiwan; 6 Department of Urology, Ditmanson Medical Foundation Chia-Yi Christian Hospital, Chiayi, Taiwan; 7 Department of Laboratory Medicine, Ditmanson Medical Foundation Chia-Yi Christian Hospital, Chiayi, Taiwan; 8 Department of Internal Medicine, National Taiwan University Hospital, Taipei, Taiwan; 9 Epigenomics and Human Diseases Research Center, National Chung Cheng University, Chiayi, Taiwan

**Keywords:** Microbiology, Applied microbiology, Microbial communities, Health care, Dentistry

## Abstract

Sjögren’s syndrome (SS) is a prevalent systemic autoimmune disease with substantial impacts on women’s health worldwide. Although oral *Haemophilus parainfluenzae* is reduced in SS, its significance remains unclear. This study aimed to elucidate the pathophysiological role of *H. parainfluenzae* in SS. Reduced salivary *H. parainfluenzae* levels in SS patients were confirmed through quantitative PCR. Oral *H. parainfluenzae* inoculation in NOD mice alleviated focal sialadenitis, improved salivary function, and reduced IFN-γ^+^CD3^+^ and IFN-γ^+^CD8^+^ T cells in salivary gland-draining lymph nodes, maintaining immune homeostasis against a biased type 1 response. Inoculation also enhanced salivary microbiota diversity, balanced the Firmicutes-to-Proteobacteria ratio, and reduced the overwhelming presence of *Pseudomonas mendocina*. In vitro, *H. parainfluenzae*-preconditioned A253 cells limited CD8 T cell expansion with reduced IFN-γ production. These findings suggest that *H. parainfluenzae* improves oral microbial diversity, promotes homeostatic T-cell immunity, and protects against SS, supporting its potential as a next-generation probiotic.

## Introduction

Sjögren’s syndrome (SS) is a systemic autoimmune disease characterized by inflammation and dysfunction of the exocrine glands. Beyond this, SS often presents with extra glandular manifestations affecting multiple organs and systems^1^ and poses an increased risk of certain malignancies^2^. As a result, SS impairs quality of life and frequently leads to work-related disability^3^. With a strong preference to affect perimenopausal women, SS greatly impacts women’s health worldwide^4^.

To date, the treatment of SS remains limited due to its complex etiology. The pathogenesis of SS involves a multifaceted interplay between genetic predispositions and environmental factors. Epithelial cells, dendritic cells, B cells, and T cells contribute to this process by producing a wide array of cytokines, chemokines, growth factors, and antibodies, ultimately leading to the destruction of exocrine glands^5^. Among these immune cells, T cells are of particular interest as they constitute the predominant inflammatory infiltrates in focal sialadenitis, a hallmark histopathological feature of SS^6^. These infiltrates often exhibit a bias towards type 1 immunity, characterized by the production of IFN-γ^6^.

IFN-γ, produced primarily by T cells, is the signature cytokine of type 1 immunity and plays an indispensable role in cellular immunity. However, it also appears to participate in the pathogenesis of SS by activating salivary gland epithelial cells. This activation leads to enhanced production of proinflammatory cytokines and chemokines, aberrant expression of MHC class II and co-stimulatory molecules, induction of apoptosis, and impairment of epithelial integrity^7^. Moreover, mice deficient in IFN-γ or its receptor are protected from SS-like disease, highlighting the essential role of IFN-γ in the pathogenesis of SS^8^.

The microbiota contributes to the health of its host^9^. It shapes the host’s immune system, and disruptions in the microbiota increase the host’s vulnerability to immune-mediated diseases^10^. While a minority of the microbiota is considered pathobionts, the majority consists of innocuous commensals that actively contribute to the host’s immune homeostasis^10^. These commensals, including segmented-filamentous bacteria found in the gut of mice, maintain CD4 T cell immunity through a unique process of delivering cell wall antigenic components to host cells, thereby preventing experimental *Citrobacter* infection in the host^11,12^. Additionally, the ubiquitous gut commensal *Bacteroides fragilis* regulates the CD4 T cells towards more balanced type 1 and type 2 immune responses and promotes functional regulatory T cells via the outer membrane component polysaccharide A in a TLR2-dependent manner^13–16^.

Several additional reports highlight the role of gut microbiota in host immune homeostasis^17^. For instance, colonization by Clostridia clusters IV, XIVa, and XVIII derived from the human gut microbiota limits abberant Th2 and Th17 immune responses^18^. Gut microbial metabolites, particularly butyrate with its histone deacetylase-inhibitory activity, reduce proinflammatory cytokines in dendritic cells^19^. In summary, the gut microbiota promotes host immune homeostasis by engaging host cells through its structural components or metabolites.

On the contrary, the contribution of oral microbiota to host immune homeostasis remains largely unexplored. The oral cavity houses over 600 bacterial species and is considered a common source for gut and airway microbiota^20,21^. Certain oral species, like *Porphyromonas gingivalis* and *Aggregatibacter actinomycetemcomitans*, have been linked to rheumatoid arthritis, contributing to autoimmunity^22^. *Prevotella melaninogenica*, enriched in the salivary microbiota of SS patients, upregulates MHC molecules, CD80, and type III IFN in salivary gland epithelial cells and is readily detectable in association with focal sialadenitis^23^.

In contrast, oral *Haemophilus parainfluenzae* is depleted in various autoimmune and chronic inflammatory diseases^23–26^. In our previous study, *H. parainfluenzae* was the most depleted species in the salivary microbiota of SS patients, as demonstrated by the greatest effect size at the species level^24^. Moreover, *H. parainfluenzae*-preconditioned salivary gland epithelial cells limited CD4 T cell expansion^24^. Therefore, we propose an immunomodulatory and protective role of *H. parainfluenzae* in SS. To investigate this hypothesis, the non-obese diabetic (NOD) mouse was utilized in this study.

The NOD mouse is a well-characterized animal model of polyautoimmunity with dysregulated T-cell immunity^27^. In addition to autoimmune diabetes, NOD mice spontaneously develop focal sialadenitis with salivary dysfunction, resembling human SS^28^. Notably, this sialadenitis predominantly involves T cells, similar to observations in human subjects^6,29^. Moreover, sialadenitis in either NOD mice or SS patients exhibits a biased immune response towards type 1 immunity^6,30^.

This study investigates the hypothesis that *H. parainfluenzae* confers protection against SS and contributes to maintaining host immune homeostasis. Additionally, it examines whether *H. parainfluenzae* improves oral microbiota, given the widespread presence of oral dysbiosis in SS patients^31^. Initially, a reduction in salivary *H. parainfluenzae* among subjects with SS was confirmed through qPCR. Subsequent oral inoculation of NOD mice with *H. parainfluenzae* led to alleviated disease and resistance to a biased type 1 immune response, indicating maintenance of homeostatic immunity. Additionally, *H. parainfluenzae* improved host salivary microbiota towards enhanced microbial diversity and a more balanced Firmicutes-to-Proteobacteria ratio. Lastly, *H. parainfluenzae* attenuated the type 1 immune response in vitro using human cells. Collectively, this study suggests a protective effect of *H. parainfluenzae* in SS and highlights its promising role in upholding host immune homeostasis and regulating the oral microbiota.

## Results

### Decreased salivary *H. parainfluenzae* in SS patients

**Fig. 1:**
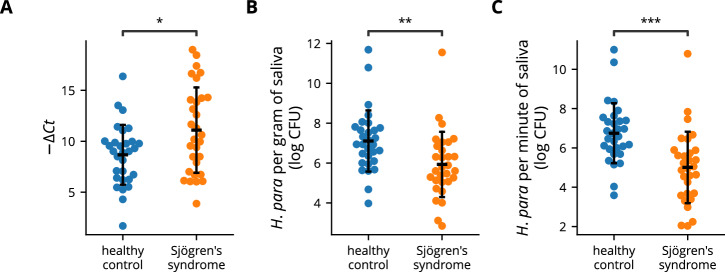
Decreased abundance of salivary *H*. *parainfluenzae* in Sjögren’s syndrome patients. Salivary DNA from Sjögren’s syndrome patients (n = 30) and healthy controls (n = 30) was analyzed using qPCR. **A** A swarm plot depicting the difference in *Ct* values obtained with *H. parainfluenzae*-specific and universal eubacteria primers. A greater −Δ*Ct* value indicates lower relative abundance. **B** Salivary *H. parainfluenzae* concentration was calculated by sample weight and the *Ct* values obtained with *H. parainfluenzae*-specific primers. **C** Total salivary *H. parainfluenzae* was defined as CFU per minute of saliva production and was calculated using the unstimulated whole salivary flow rate, sample weight, and the *Ct* values obtained with *H. parainfluenzae*-specific primers. Statistical significance was determined by Student’s *t-*test (*p < 0.05, **p < 0.01, ***p < 0.001). Error bars represent standard deviation. *H. para*: *Haemophilus parainfluenzae*.

Oral *H. parainfluenzae* accounts for the majority of oral *Haemophilus*^24,32^. Previous studies conducted 16S ribosomal sequencing, revealing reduced oral *Haemophilus* or *H. parainfluenzae* in individuals with SS^23,24,31^; however, there are also negative reports^31^. To confirm whether oral *H. parainfluenzae* is reduced in SS, qPCR using *H. parainfluenzae*-specific primers and universal primers was conducted to evaluate *H. parainfluenzae* abundance in saliva samples from 30 SS patients and 30 healthy controls (Table 1). The Δ*Ct* between *H. parainfluenzae-*specific and universal primers was greater among SS patients, indicating a decreased relative abundance of salivary *H. parainfluenzae* (Fig. 1A). In addition, the salivary concentration of *H. parainfluenzae*, represented as CFU per gram of saliva (Fig. 1B), and the total salivary *H. parainfluenzae*, estimated as CFU per minute of saliva production (Fig. 1C), were markedly reduced in individuals with SS. These findings affirm the prior discovery of reduced oral *H. parainfluenzae* relative abundance in SS patients using 16S ribosomal sequencing and provide insights into the diminished concentration and total amount of salivary *H. parainfluenzae* in SS. The low number of male participants limited further sex-based analysis.

**Table 1:** Baseline characteristics of study participants

	healthy controls (*n* = 30)	Sjögren’s syndrome (*n* = 30)
age (year, mean ± SD)	51.6 ± 10.2	51.3 ± 10.2
women (*n*, %)	28 (93)	29 (97)
smoking (*n*, %)	0 (0)	0 (0)
‍comorbid disease (*n*, %)		
‍ hypertension	5 (17)	4 (13)
‍ diabetes mellitus	0 (0)	0 (0)
‍ chronic kidney disease	0 (0)	0 (0)
‍ liver cirrhosis	0 (0)	1 (3)
‍ autoimmune disease (including Sjögren’s syndrome)	0 (0)	30 (100)
duration of xerostomia (year, mean ± SD)	0 ± 0	5.6 ± 4.5
unstimulated whole salivary flow rate (ml/min, mean ± SD)	0.50 ± 0.28	0.17 ± 0.12
hyposalivation (*n*, %)	0 (0)	11 (37)
Schirmer’s test ≤ 5 mm (*n*, %)	NA	29 (97)
ESSDAI (point, mean ± SD)	NA	4.4 ± 2.8
anti-Ro positivity (*n*, %)	NA	20 (67)
IgG (mg/dL, mean ± SD)	NA	1481 ± 541
received LSG biopsy with focus score ≥ 1 (*n*, %)	NA	10 (33)
medication (*n*, %)		
hydroxychloroquine	0 (0)	17 (57)
sulfasalazine	0 (0)	2 (7)
methotrexate	0 (0)	2 (7)
cyclosporin	0 (0)	1 (3)
azathioprine	0 (0)	2 (7)
corticosteroids	0 (0)	7 (23)
secretagogues	0 (0)	6 (20)

*SD* standard deviation, *NA* not applicable, *ESSDAI* European League Against Rheumatism Sjögren’s syndrome disease activity index, *LSG* labial salivary gland.

### *H. parainfluenzae* alleviates SS-like disease and modulates T cells in NOD mice

**Fig. 2:**
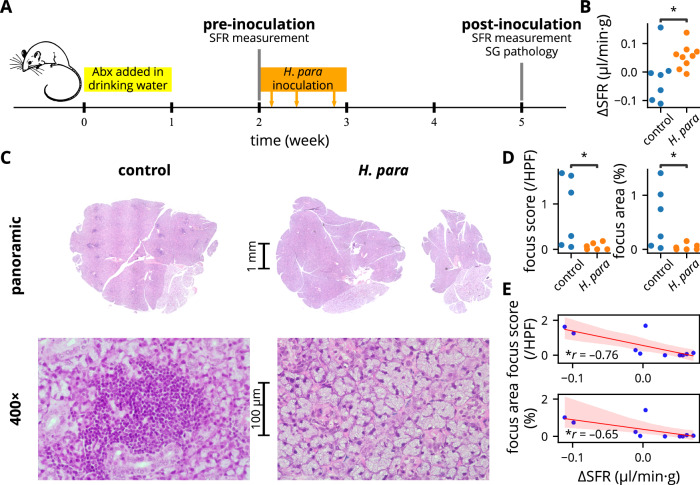
*H*. *parainfluenzae* inoculation ameliorates Sjögren’s syndrome-like disease in NOD mice. **A** Summarized experiemental procedure. Mice were randomly allocated to either the control or *H. parainfluenzae* group in alternating sequence after the first SFR measurement. Pilocarpine preparation used for SFR measurement and *H. parainfluenzae* preparation for inoculation were both derived from the same batch. **B** Change in SFR normalized by body weight, with statistical significance determined using Student’s *t* test. Of a total of 22 mice (11 mice for each group), SFR measurement failed in two and four mice at baseline and after *H. parainfluenzae* inoculation respectively. These mice were excluded from the analysis. **C** Representative salivary gland histology by H&E stain. Salivary glands were harvested from six randomly selected mice in each group. Focal sialadenitides were represented by dark-stained areas in the panoramic view showing mononuclear aggregates (see 400× magnification), evident in the control mouse but almost absent in the *H*. *parainfluenzae*-inoculated mouse. **D** Focus score and focus area analysis with statistical significance determined using the Mann-Whitney U test. **E** Regression plot between the degree of focal sialadenitis and change in SFR, with Pearson’s correlation coefficient presented. Abx: antibiotic, SFR: salivary flow rate, SG: salivary gland, *H. para*: *Haemophilus parainfluenzae*, *p < 0.05.

NOD mice spontaneously develop focal sialadenitis with decreased salivation, similar to SS patients^28^. Since SS preferentially affects women^4^, this study focused exclusively on female mice. NOD mice were orally inoculated with *H. parainfluenzae* and analyzed for changes in salivary flow rate and focal sialadenitis (experimental procedure shown in Fig. 2A). Salivary flow rate improved in *H. parainfluenzae-*inoculated mice (Fig. 2B). Correspondingly, these mice exhibited a lower degree of focal sialadenitis, as demonstrated by a lower focus score and focus area (Fig. 2C, D). The change in salivary flow rate negatively correlated with both the focus score and focus area (Fig. 2E). These findings collectively suggest a disease-ameliorating effect of oral *H. parainfluenzae* inoculation.

**Fig. 3:**
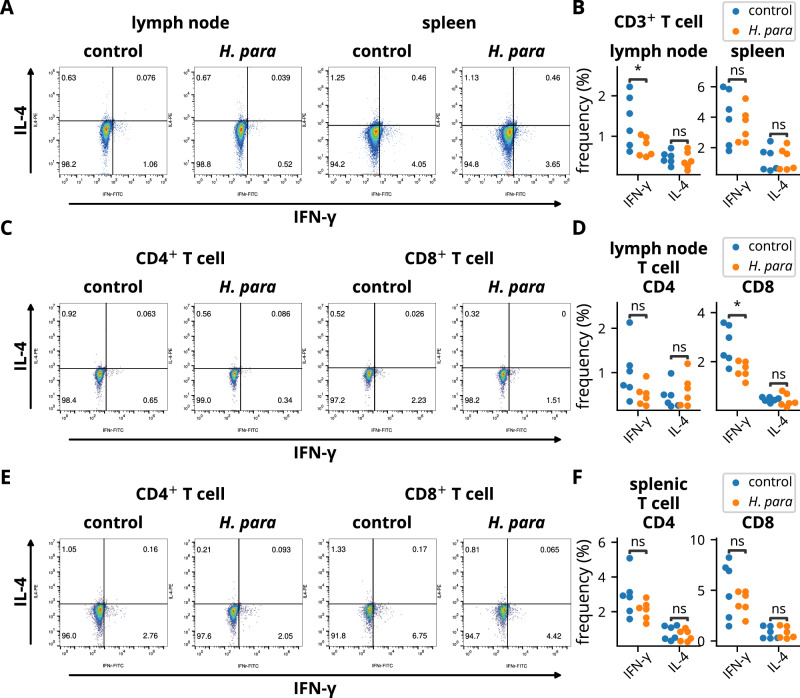
Immunomodulatory effect of *H*. *parainfluenzae* inoculation on T cells. Cells from the salivary gland-draining lymph nodes and spleens of six randomly selected NOD mice in each group were harvested two weeks after the completion of *H. parainfluenzae* inoculation and subjected to flow cytometry. **A**, **B** Cells were gated on CD3 and stained for IFN-γ and IL-4. Representative dot plots and statistical analyses are shown. **C**–**F** Cells were gated on CD3 and CD4 or CD8 and stained for IFN-γ and IL-4. Representative dot plots and statistical analyses of salivary gland-draining lymph nodes (**C-D**) or splenic T cells (**E-F**) are presented. Statistical significance was determined by Student’s *t-*test (*p < 0.05). *H. para*: *Haemophilus parainfluenzae*.

Since IFN-γ plays a critical role in the pathogenesis of SS-like disease in NOD mice^8^, T cells from salivary gland-draining lymph nodes and spleen were detected for IFN-γ and IL-4 expression. IFN-γ expression was reduced in T cells from the salivary gland-draining lymph nodes (Fig. 3A, B). A trend towards a reduction of IFN-γ^+^ CD4^+^ T cells (*p* = 0.089) and a reduction of IFN-γ^+^ CD8^+^ T cells was demonstrated (Fig. 3C, D). In contrast, IFN-γ expression were not reduced in CD3^+^, CD4^+^, or CD8^+^ T cells of the spleen (Fig. 3A–F), indicating a localized immunomodulatory effect towards an attenuated type 1 immune response in T cells. IL-4 expression was not altered (Fig. 3A–F). Overall, these results suggest resistance to a biased type 1 immune response in the draining lymph nodes of *H. parainfluenzae*-inoculated mice, which correlates with the observed disease-ameliorating effect.

### *H. parainfluenzae* promotes diversity and resilience of the oral microbiota

**Fig. 4:**
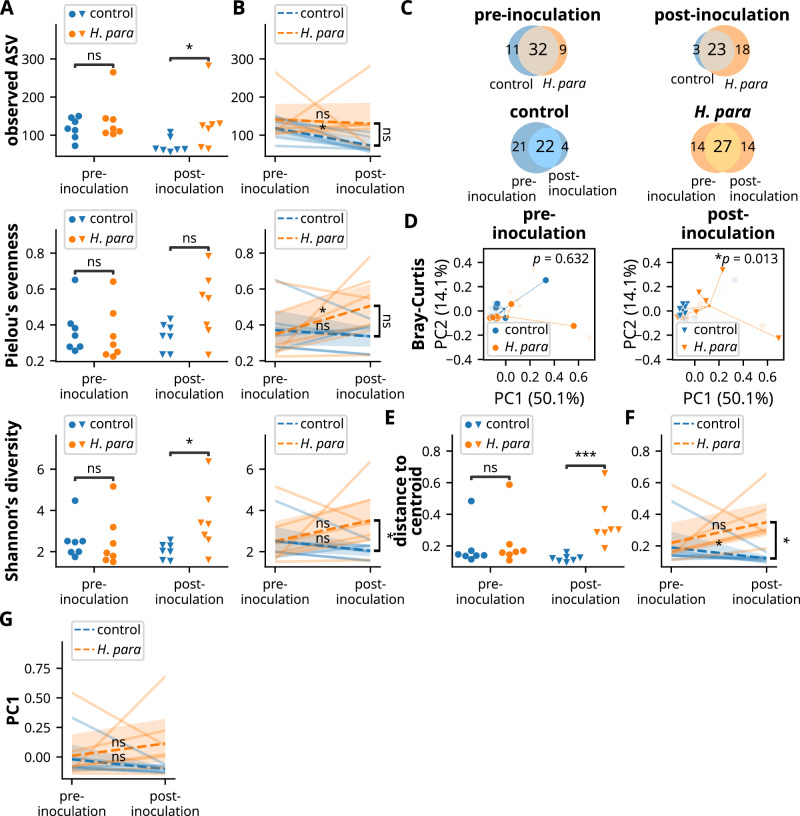
*H*. *parainfluenzae* inoculation improves oral microbial diversity and resilience in NOD mice. Since paired saliva samples (pre- and post-inoculation) were available from seven control mice, an additional seven paired saliva samples were randomly selected from *H. parainfluenzae*-inoculated mice. Saliva was then subjected to 16S ribosomal sequencing, with diversity computed based on amplicon sequencing variants (ASVs). Statistical significance was determined using the Mann-Whitney U test in non-paired analysis whenever not otherwise indicated. The Wilcoxon rank-sum test was utilized for paired analysis within individual groups, and the Mann-Whitney U test was used for comparison of paired changes between control and *H. parainfluenzae*-inoculated mice. **A**, **B** Species richness, evenness, and diversity of salivary microbiota in unpaired (**A**) and paired (**B**) analyses. **C** Venn diagrams depicting common and unique core ASVs. **D** Principal coordinate analysis utilizing Bray-Curtis distance showing compositional differences. The central point indicates the centroid of each group. Statistical significance was determined by PERMANOVA. **E**, **F** Within-group dispersion was evaluated by distance to the group centroid of principal coordinates. Unpaired (**E**) and paired (**F**) analyses. **G** Paired analysis on PC1. *H. para*: *Haemophilus parainfluenzae*, *p < 0.05, ***p < 0.001.

To evaluate whether oral *H. parainfluenzae* inoculation led to improvement in oral microbiota, the salivary microbiota of NOD mice was profiled using 16S ribosomal sequencing. Oral *H. parainfluenzae* inoculation led to increased species richness, a trend toward increased evenness, and increased diversity at the ASV level (Fig. 4A). In paired analyses for pre-inoculation and post-inoculation salivary samples from the same mouse, species richness decreased in control mice but remained stable in *H. parainfluenzae-*inoculated mice (Fig. 4B). Evenness increased in *H. parainfluenzae-*inoculated mice but not in control mice, with a notable trend toward divergent evenness (*p* = 0.073, Fig. 4B). Although diversity did not change significantly in either group according to paired analysis, there were significant divergences between the groups (Fig. 4B), indicating an effect of inoculation on diversity.

Analysis of the core microbiota, defined by observed ASVs in more than half of the samples in each subgroup, also corresponds to the observation of enhanced diversity. A Venn diagram illustrates reduced numbers of total core ASVs and unique core ASVs in control mice (Fig. 4C). Before inoculation, there were 43 and 41 core ASVs in control and *H. parainfluenzae*-inoculated mice respectively and 26 and 41 core ASVs post-inoculation (Fig. 4C). Addtionally, there were fewer unique core ASVs in control mice: three and 18 unique core ASVs in control and *H. parainfluenzae*-inoculated mice, respectively, or four unique core ASVs in control and 14 unique core ASVs in *H. parainfluenzae*-inoculated mice compared to their pre-inoculation counterparts (Fig. 4C). These results indicate an effect of *H. parainfluenzae* inoculation toward enhanced alpha-diversity.

Consistent with differences in alpha-diversity, the salivary microbiota significantly differed after *H. parainfluenzae* inoculation, as shown by principal coordinate analysis (Fig. 4D). Notably, the salivary microbiota in control mice became more converged (Fig. 4D), demonstrated by decreased intragroup dispersion (Fig. 4E). In paired analysis, declining intragroup dispersion was evident in the control mice, but not in the *H. parainfluenzae*-inoculated mice, showing a significant difference between groups (Fig. 4F). To determine whether this maintenance of intragroup dispersion in *H. parainfluenzae*-inoculated mice was associated with enhanced microbial resilience, a paired analysis on PC1, which accounted for 50.1% of the principal component (Fig. 4D), revealed a notable trend of change in control mice (*p* = 0.078), but not in *H. parainfluenzae*-inoculated mice (*p* = 0.297, Fig. 4G). Overall, these results indicate a resistance to a convergent microbiota in *H. parainfluenzae-*inoculated mice, suggesting a possible role of *H. parainfluenzae* in the maintenance of microbial resilience.

### *H. parainfluenzae* inoculation leads to reduced salivary Proteobacteria and *Pseudomonas mendocina*

**Fig. 5:**
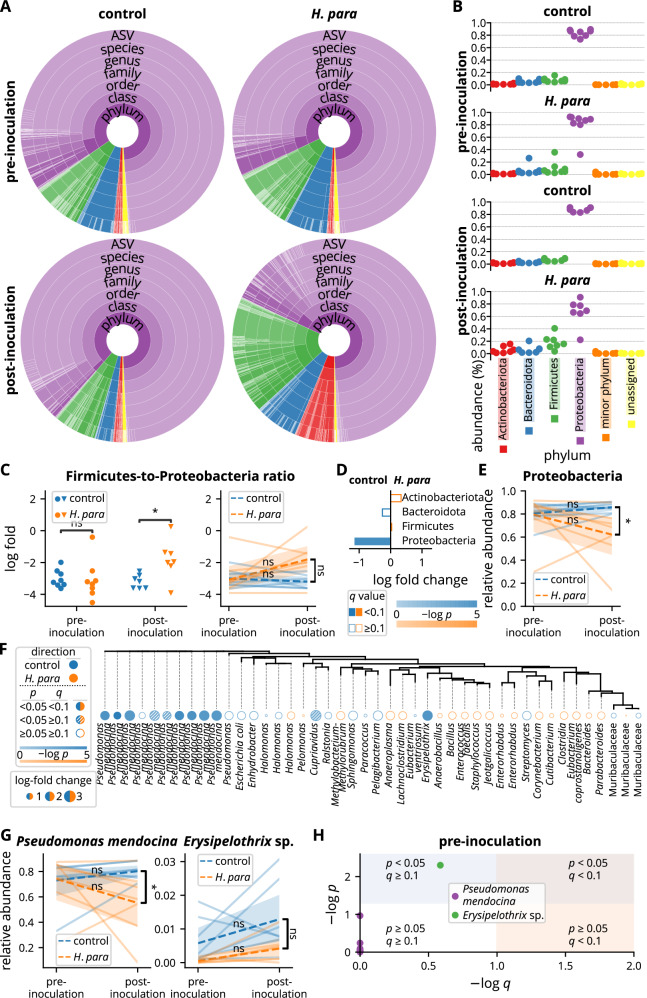
Reduced salivary Proteobacteria and *Pseudomonas mendocina* following *H. parainfluenzae* inoculation. **A** Summarized salivary microbiota. Colors correspond to taxa belonging to phyla as indicated in **B**. Each color depth corresponds to an individual taxon. The arc angle represents relative abundance. **B** Relative salivary abundance at the phylum level. **C** Analyses on Firmicutes-to-Proteobacteria ratio. The y-axis represents log fold with the base of *e*. Statistical significance was determined using the Mann-Whitney U test or the Wilcoxon rank sum test. **D** Comparison at the phylum level analyzed by ANCOM-BC. **E** Paired analysis of Proteobacteria abundance with statistical significance determined using the Wilcoxon rank sum test or the Mann-Whitney U test. **F** Comparison at the ASV level by ANCOM-BC. The tree was built based on phylogenetic similarity. Each tree label indicates the lowest annotated taxon of an ASV. **G** Paired analyses of *Pseudomonas mendocina* and the differentially abundant ASV annotated as *Erysipelothrix* sp. in **F**. Statistical significance was determined using the Wilcoxon rank sum test or the Mann-Whitney U test. **H** Analysis of the pre-inoculational abundance of ASVs annotated to *Pseudomonas mendocina* and the differentially abundant ASV annotated as *Erysipelothrix* sp. in **F** by ANCOM-BC. ASV amplicon sequencing variant, *H. para*: *Haemophilus parainfluenzae*, *p < 0.05.

The salivary microbiota was further analyzed at the phylum level. Actinobacteriota, Bacteroidota, Firmicutes, and Proteobacteria had mean relative abundances greater than 1%, comprising the majority of the salivary microbiota (Fig. 5A, B). Proteobacteria dominated the salivary microbiota, followed by Firmicutes, Bacteroidota, and Actinobacteriota (Fig. 5A, B). The dominance of Proteobacteria was attributed to a single dominant ASV (Fig. 5A). The microbiota became more balanced in *H. parainfluenzae*-inoculated mice, with the Firmicutes-to-Proteobacteria log fold ratio closer to zero (Fig. 5C). A trend toward a divergent Firmicutes-to-Proteobacteria ratio persisted in paired analysis (*p* = 0.053, Fig. 5C). Analysis by ANCOM-BC confirmed a significant depletion of Proteobacteria but not an increase in Firmicutes, in *H. parainfluenzae*-inoculated mice (*p* < 0.001, *q* = 0.024, log fold change 1.14, Fig. 5D), a difference that remained evident in paired analysis (Fig. 5E).

To identify the effect of oral *H. parainfluenzae* inoculation on the salivary microbiota at lower phylogenetic levels, differential abundance was analyzed at the ASV level by ANCOM-BC. Several ASVs annotated to *Pseudomonas mendocina* exhibited significant decreases in *H. parainfluenzae*-inoculated mice (Fig. 5F). The most abundant ASV, accounting for more than half of the salivary microbiota (Fig. 5A), was also annotated to *P*. *mendocina*, showing a significant depletion in *H. parainfluenzae*-inoculated mice (*p* < 0.001, *q* = 0.001, log fold change 1.59, Fig. 5F). The depletion of *P*. *mendocina* ASVs in *H. parainfluenzae*-inoculated mice was further confirmed in the analysis at the species level, which showed decreased *P*. *mendocina* abundance in paired analysis, demonstrating divergent abundance changes (Fig. 5G). Although an ASV annotated to an *Erysipelothrix* sp. was also differentially abundant with ANCOM-BC (*p* = 0.001, *q* = 0.048, log fold change 2.94, Fig. 5F), there was no significant difference in paired analysis (Fig. 5G). Further analysis revealed a depletion of this ASV pre-inoculationally with a *p* value of 0.005 but a *q* value of 0.259 (Fig. 5H), suggesting a depletion at baseline that might be attributed to chance. In contrast, none of the ASVs annotated to *P*. *mendocina* had a *p* value less than 0.05 pre-inoculationally (Fig. 5H). Overall, *H. parainfluenzae* inoculation resulted in a depletion of Proteobacteria at the phylum level and *P*. *mendocina* at the species level.

### *H. parainfluenzae*-preconditioned salivary gland epithelial cells limit CD8 T cell expansion with attenuated type 1 immune response

**Fig. 6:**
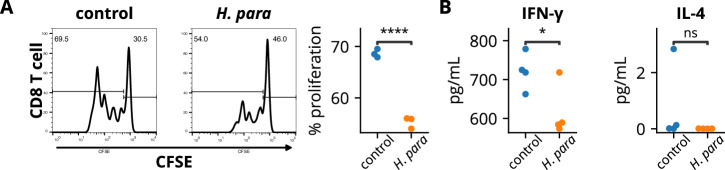
*H. parainfluenzae*-pre conditioned A253 cells suppressed CD8 T cell proliferation with reduced IFN-γ production. A253 cells preconditioned with heat-inactivated *H. parainfluenzae* at a bacteria-to-cell ratio of 100:1 were cocultured with CD8 T cells. **A** Cells undergoing proliferation exhibited diminished fluorescent intensity of carboxyfluorescein succinimidyl ester (CFSE). The percentage of CD8 T cells undergoing proliferation was analyzed. **B** Supernatant cytokine assay. Statistical significance was determined using the Student’s *t-*test. *H. para*: *Haemophilus parainfluenzae*, *p < 0.05, ****p < 0.0001.

To validate the immunomodulatory effect of *H. parainfluenzae* on human CD8 T cells, A253 cells, a human salivary gland cell line with epithelial morphology^33^, were preconditioned with *H. parainfluenzae* and cocultured with human CD8 T cells. The *H. parainfluenzae*-preconditioned A253 cells suppressed CD8 T cell proliferation (Fig. 6A) and decreased IFN-γ levels in the co-culture supernatant (Fig. 6B). These results correspond to the finding of decreased IFN-γ-producing CD8^+^ T cell in *H. parainfluenzae*-inoculated mice (Fig. 3C, D). No difference in supernatant IL-4 levels was detected (Fig. 6B). These results support the modulatory effect of *H. parainfluenzae* on the CD8 T cell response in human cells, similar to the in vivo results observed in NOD mice.

## Discussion

We previously proposed a role of *H. parainfluenzae* in maintaining host immune homeostasis^24^. The present study confirms a reduction in salivary *H. parainfluenzae* levels in patients with SS, as demonstrated by quantitative PCR. In a murine model, NOD mice inoculated with *H. parainfluenzae* exhibited mitigated SS-like disease, enhanced oral microbial diversity and probably resilience, and a decrease in IFN-γ-producing CD8^+^ T cells. Additionally, human CD8 T cell expansion was limited by *H. parainfluenzae*-preconditioned A253 cells, which were associated with lower levels of IFN-γ in the supernatant. These findings highlight the consistent effect of *H. parainfluenzae* in both animal models and human cells, emphasizing its role in the maintenance of homeostatic immunity against a biased type 1 immune response, oral microbiota regulation, and protection against SS.

The current study validated reports showing a decrease in *H. parainfluenzae* relative abundance in the oral microbiota of SS patients through qPCR^23,24,34^. Furthermore, a true deficiency was identified, as evidenced by significantly lower salivary concentrations and total minute amounts. The salivary concentration of *H. parainfluenzae* in healthy controls was approximately 10 times higher than in SS patients (Fig. 1B), while the total minute salivary amount was about 100 times greater (Fig. 1C). This exponential difference likely results in substantially reduced exposure to *H. parainfluenzae* in SS patients, thereby disrupting homeostatic control and potentially contributing to biased type 1 immunity.

Oral inoculation of *H. parainfluenzae* in NOD mice significantly improved salivary flow rate, which correlated with reduced salivary gland inflammation. These findings provide proof of concept for the active participation of the oral microbiota in SS pathogenesis, rather than merely being a passive response to changes in the oral microenvironment. In addition to *H. parainfluenzae*, several phylogenetically related taxa, such as the genus *Neisseria*, which also belongs to the class Gammaproteobacteria, have also been reported as depleted in the oral microbiota of SS patients^31,34^. These taxa may contribute to protection against SS, warranting further investigation.

The present study highlights the role of *H. parainfluenzae* in the maintainence of host homeostatic immunity against a biased type 1 immune response. Oral inoculation reduced IFN-γ-producing T cells in the salivary gland-draining lymph nodes of NOD mice but not in splenic T cells, suggesting a localized effect sufficient to ameliorate disease. The relatively lower administration dose, compared to those used in gut microbiota research, likely contributed to this observation. Additionally, *H. parainfluenzae*-preconditioned salivary gland epithelial cells limited CD8 T cell expansion and reduced IFN-γ production, showing a critical role of microbiota-epithelial-immune cell cross-talk.

In contrast to the attenuated type 1 immune response, IL-4 levels were not changed. As a signature type 2 cytokine, IL-4 has been implicated in SS pathogenesis, particularly in cases with evident ectopic germinal centers or those complicated by lymphoma, a known late-stage complication of SS^35^. The finding in this study may reflect the short observation period, warranting further investigation.

The present study demonstrated that *H. parainfluenzae* plays a role in maintaining T cell immune homeostasis, particularly with regard to CD8 T cells. In autoimmune diseases, CD8 T cells can break self-tolerance and exhibit inappropriate effector functions, leading to tissue damage^36^. CD8 T cells are increasingly recognized in the pathogenesis of SS^37–39^. Tissue-resident memory CD8 T cells are expanded in the salivary glands of SS patients, exhibiting IFN-γ production, while depletion of CD8 T cells or in situ tissue-resident memory CD8 T cells protects mice from SS-like disease^39,40^. The current study provides a novel perspective of CD8 T cell regulation by oral commensals. A more comprehensive experimental design to elucidate the effects of *H. parainfluenzae* on CD8 T cells would be valuable.

Combined with its demonstrated immunomodulatory effects in vivo and in vitro, these results suggest a direct protective role of *H. parainfluenzae* in SS. Dendritic cells, the most potent antigen-presenting cells, activate T cells in the lymphoid organs by presenting antigens acquired peripherally, providing co-stimulatory signals, and producing cytokines^41^. Commensals are known to shape dendritic cells into a tolerogenic phenotype, thereby modulating T cell functions^19,42^.

Based on the findings of this study, a plausible hypothesis is that *H. parainfluenzae* primes dendritic cells in the oral cavity, directing them toward a tolerogenic phenotype. These dendritic cells may then migrate to draining lymph nodes, where they limit IFN-γ-producing T cells. Additionally, *H. parainfluenzae* may modulate salivary gland epithelial cells to restrict in situ CD8 T cell expansion, particularly tissue-resident memory CD8 T cells^39,40^, leading to reduced IFN-γ production. However, the possibility of a direct effect of *H. parainfluenzae* on T cells, or its modulation through other cell types, requires further investigation.

The oral microbiota changes following *H. parainfluenzae* inoculation reflect several findings from human oral microbiota studies in SS: the increased microbial diversity in *H. parainfluenzae*-inoculated mice parallels reports of higher diversity in healthy controls^34,43–45^; the decrease in unique ASVs in control mice corresponds with findings of reduced unique ASVs or genera in SS patients^34,46^; and the more balanced Firmicutes-to-Proteobacteria ratio after *H. parainfluenzae* inoculation is consistent with observations in healthy controls^34,47^. Moreover, *H. parainfluenzae* inoculation led to resistance to a convergent microbiota, suggesting a more durable microbiota with potentially enhanced resilience. These findings suggest a more diversified oral environment and/or a more balanced immune system following *H. parainfluenzae* inoculation or in healthy controls, although direct interspecific interactions cannot be excluded and require further study.

Another interesting finding is the overwhelming presence of *P. mendocina* in the salivary microbiota of NOD mice. A previous study demonstrated a similar predominance of Proteobacteria in oral swabs from NOD mice, with Proteobacteria predominantly occupied by a single genus, *Aggregatibacter* followed by *Mannheimia*^48^. In contrast, *Pseudomonas* predominated in the present study. It is likely that specific host factors in NOD mice facilitate a monotonous oral microbiota, with the dominant species being determined by housing conditions. Further research on these topics may be of interest given the scarcity of reports on the oral microbiota of NOD mice.

This study also provides provisional insights into the potential of *H. parainfluenzae* as a next-generation probiotic. Next-generation probiotics, unlike conventional probiotics (primarily limited to *Lactobacillus* and *Bifidobacterium* spp.), utilize microbes not traditionally associated with promoting human health^49^. Developing *H. parainfluenzae* into clinical application should also include comprehensive in vitro and in vivo evaluations of its related bio-products. These bio-products, or postbiotics, include, but are not limited to, culture supernatants, dead or inactivated bacteria, and fractionated bacterial components^50^. Similar to other Gram-negative bacteria, *H. parainfluenzae* has been observed to release outer membrane vesicles with compositions resembling its outer membrane^51,52^. The isolation and further application of these outer membrane vesicles are also areas of interest for advancing its potential as a source of postbiotics.

For preclinical studies, various disease models focusing on treatment and prevention may be utilized, based on prior findings of reduced oral *Haemophilus* or *H. parainfluenzae* in several diseases associated with inflammation and autoimmunity^25,26,34,53–57^. For clinical translationality, *H. parainfluenzae* or its related bio-products could be formulated into chewing gum, toothpaste, or mouthwash, while a gel-based application would potentially offer enhanced oral retention. Another area of interest could involve exploring mechanisms and strategies to maintain or enhance the abundance of oral *H. parainfluenzae*.

The major limitation of this study arises from the significant difference between the oral microbiota of humans and NOD mice, which are addressed as follows:

First, there are differences in the represented taxa. At the phylum level, the oral microbiota is represented by Firmicutes, followed by Proteobacteria in human subjects, but Proteobacteria followed by Firmicutes in NOD mice. *Streptococcus* is the most abundant genus in human oral microbiota; in contrast, *Pseudomonas* in this study or *Aggregatibacter* are predominant in NOD mice^48^. It remains unclear whether these background microbiota differences impact interspecific interactions and study outcomes.

Second, the oral microbiota of NOD mice exhibits a monotonous composition, which is considered more vulnerable and less resilient^58^. For example, in NOD mice, the oral microbiota is predominantly represented by one or two genera or a single ASV. This simplified microbiota may be more sensitive to environmental changes, such as *H. parainfluenzae* inoculation, potentially leading to amplified effects. In contrast, the human oral microbiota is significantly more diverse, offering a buffer against such environmental changes. Consequently, whether the effects of *H. parainfluenzae* observed in this study can be directly extrapolated to human subjects remains uncertain.

Lastly, while *H. parainfluenzae* is ubiquitously present in the human oral microbiota, it is absent in NOD mice (data not shown). Although this difference may limit the generalization of the findings to humans, the absence of *H. parainfluenzae* in NOD mice provided a clean microbiota background, minimizing variability associated with baseline *H. parainfluenzae* levels.

There are additional considerations in interpreting the study results. NOD mice spontaneously develop autoimmune destruction of pancreatic β cells^59^. Although all mice were non-diabetic at the time of sacrifice, it remains uncertain whether β cell loss at the prediabetic stage may have confounded the findings of the current study. Another notable difference is the age of disease onset: while SS predominantly affects peri-menopausal women, NOD mice exhibit an earlier onset of SS-like disease^60^. These factors highlight the need for caution when extrapolating the results to human cases.

In summary, *H. parainfluenzae* protects individuals from SS with improved oral microbial diversity and probably enhanced resilience. This study also provided sufficient evidence to support the contribution of *H. parainfluenzae* to T cell immune homeostasis, demonstrating resistance to a biased type 1 immune response. Further research is required to elucidate more detailed mechanisms and explore the potential applicability of *H. parainfluenzae* as a next-generation probiotic.

## Methods

### Study participants and saliva collection

SS patients attending the rheumatology clinic at the Ditmanson Medical Foundation Chia-Yi Christian Hospital (Chiayi, Taiwan), who met the 2016 ACR/EULAR Classification Criteria for Primary Sjögren’s Syndrome, were enrolled^61^. Healthy controls with no prior diagnosis of autoimmune disease were recruited from the community. Smokers and individuals with diabetes were excluded due to their potential confounding on oral *Haemophilus* or *H. parainfluenzae*^62,63^. Unstimulated whole saliva was collected as previously described^24^, with simultaneous measurement of salivary flow rate, a criterion in the classification of SS^61^.

In our previous study, we identified a depletion of salivary *H. parainfluenzae* in Sjögren’s syndrome patients using 16S ribosomal sequencing of saliva samples from 24 individuals who had not recently used corticosteroids or medications for immune modulation or suppression^24^. For this study, SS patients with recent use of corticosteroids, medications for immune modulation or suppression, and secretagogues were also included, and a different detection method (qPCR) was employed. The sample size was increased to 30 healthy controls and 30 Sjögren’s syndrome patients to account for the expected higher variability and uncertainty associated with the qPCR method. In summary, the demographic data of all study participants were consistent with previous reports, showing a strong predominance of perimenopausal women (Table 1)^4^.

All research procedures adhered to the revised 2013 Helsinki Declaration. This study was approved by the Research Ethics Committee of the Ditmanson Medical Foundation, Chiayi Christian Hospital (IRB: 106031), and written informed consent was obtained from all participants.

### DNA extraction and qPCR

Salivary DNA was extracted using the Genomic DNA Mini Kit (Geneaid, Taiwan), following the manufacturer’s protocol. The quantitative PCR reactions were performed on an ABI Step-One real-time PCR system (Applied Biosystems, CA). Primer sequences were adopted according to previous reports:

Universal primers^64^:

Forward: GTGSTGCAYGGYTGTCGTCA

Reverse: ACGTCRTCCMCACCTTCCTC

*H. parainfluenzae*-specific primers^26^:

Forward: ACCGTGGTCGTTTAGCAATC

Reverse: GTCCGGGTTTACGTTTAGCA

### Calculation of relative abundance, salivary *H. parainfluenzae* concentration, and total bacteria load

After obtaining the threshold cycle (*Ct*) values, the 2^−Δ*Ct*^ method was used to calculate *H. parainfluenzae* relative abundance. A smaller value denotes higher relative abundance. To estimate salivary *H. parainfluenzae* load, a calibration curve was established by qPCR for *H. parainfluenzae*-specific primers following serial dilution of a reference *H. parainfluenzae* culture. Salivary *H. parainfluenzae* concentration and total salivary *H. parainfluenzae* per minute of saliva production were calculated using the *Ct* value, saliva sample amount, and unstimulated whole salivary flow rate.

### Mice and treatment

Female NOD/ShiLtJNarl mice were purchased from the National Laboratory Animal Center (Taipei, Taiwan). A total of 22 mice (11 per group) were housed in groups of five or six per cage within microisolator cages. These mice were provided with sterilized food and water, with weekly monitoring of blood sugar levels.

During the first week, the mice were given an antibiotic cocktail in their drinking water to reduce baseline microbiota variability (Fig. 2A). The antibiotic cocktail consisted of 0.5 mg/ml ampicillin (Sigma-Aldrich), 0.5 mg/ml gentamicin (Acros Organics), and 0.25 mg/ml vancomycin hydrochloride (Sigma-Aldrich) dissolved in the drinking water. The antibiotic-containing drinking water was refreshed every other day.

After a one-week washout period, the mice were randomly assigned to either the control or treatment group in the third week. Mice in the treatment group received three doses of oral *H. parainfluenzae* inoculation (Fig. 2A). The inoculation dose was determined based on gut microbiota studies and the estimated size ratio of oral to gut microbiota, given the lack of a universal dosing standard. Doses for gut microbiota studies typically range from 10^8^ to 10^10^ CFU^65^. Based on previous estimates that the gut microbiota is approximately 1,000 times larger than the oral microbiota^66^, the dose in this study was set to 2 × 10^6^ CFU, corresponding to 20 μl of bacterial preparation at a concentration of 10^5^ CFU/μl. This volume could be safely and effectively retained in the oral cavity without causing choking or drooling. Considering the short observation period, the mice received three inoculations within a week.

To study the effect of *H. parainfluenzae* acting orally, the bacterial preparation was retained in the mouth for 30 minutes under anesthesia. The mice were placed in a prone position with their heads slightly tilted upward. The bacterial preparation was gently dispersed into the oral cavity and retained on the floor of the tongue. After 30 minutes, the mice were allowed to recover from anesthesia.

Since no prior study provided a reference, the sample size was empirically determined based on previous animal study experience. It was estimated that six to eight animals per group would be sufficient to detect significant changes. However, to account for potential failures in conducting salivary flow rate measurements, the final sample size was set to 11 animals per group.

This study adhered to the guidelines of the Institutional Animal Care and Use Committee (IACUC) and followed the “3R” principles. Approval was granted by the IACUC of National Chung Cheng University (1110907).

### Mice saliva collection and salivary flow rate measurement

Mice saliva was collected with simultaneous measurement of salivary flow rate before *H. parainfluenzae* inoculation and two weeks after completion of inoculation. Briefly, 0.225 mg/kg of pilocarpine hydrochloride (Sigma-Aldrich) was intraperitoneally administered to the mice to stimulate saliva production. Saliva was collected into a microfuge tube for 15 minutes under anesthesia by isoflurane. The amount of saliva was determined and then stored at −80 °C until analysis.

### Mice salivary gland histology and analysis of focal sialadenitis

Mice salivary glands were harvested two weeks after completion of *H. parainfluenzae* inoculation. Fixed in 10% buffered neutral formalin overnight, paraffin-embedded specimens were sectioned at 3 μm thickness and stained with hematoxylin and eosin. A focus was defined as a mononuclear aggregate containing ≥ 50 lymphoid cells. The focus score represented the number of foci per high-power field. The focus area was defined as the proportion of the area occupied by mononuclear aggregates. The evaluation of focus score and focus area was performed by an experienced pathologist blinded to the mice group.

### Fluorescent staining and flow cytometry

Cells were harvested from lymph nodes and spleen, then stained with fluorescent dye-conjugated specific antibodies at 4 °C for 30 min in the dark and subsequently washed twice with PBS. For intracellular staining, the cells were fixed and permeabilized using Cytofix/Cytoperm buffer (BD Biosciences, CA, USA) for 15 minutes prior to staining. The cells were evaluated using FACSCalibur flow cytometry, with CellQuest software (BD Biosciences), and the data were analyzed using FlowJo software (Ashland). Anti-mouse CD3-PE (145-2C11), anti-mouse CD4-APC (RM4-5), anti-mouse CD4-FITC (GK1.5), anti-mouse CD8-APC (53-6.7), anti-mouse CD8-PE (53-6.7), anti-mouse CD8-FITC (53-6.7), anti-mouse IFN-γ-FITC (XMG1.2), and anti-mouse IL-4-PE (11B11) antibodies were purchased from BD Biosciences.

### Mice saliva bacteria 16S ribosomal sequencing

Salivary DNA extraction and 16S ribosomal sequencing were processed as previously described^24^. Saliva was resuspended in PBS and subjected to centrifugation. Undissolved debris was removed by low-speed centrifugation, and the saliva was washed twice in PBS before DNA extraction. The DNA of salivary microbiota was extracted with a QIAamp DNA Stool Mini Kit (Qiagen, Hilden, Germany), following the manufacturer’s protocol. The concentration of purified DNA was determined by fluorometric spectrometry. The variable regions 3 and 4 of the bacterial 16S ribosomal DNA were amplified from the purified DNA specimens. A set of mixed primers, with one to three nucleotides placed between their annealing and adaptor sequences, was used to increase sequencing efficiency and data quality. The PCR products were separated by agarose gel electrophoresis and the expected-size products were gel-purified. A second-stage PCR using the Nextera XT index kit (Illumina Inc.) was performed to enhance sequencing efficiency. Sequencing-ready libraries were analyzed by capillary electrophoresis and quantified using a fluorescence-based method. Sequencing was performed on the MiSeq platform (Illumina Inc.) for 18 dark and 350 read cycles for the forward read and 18 dark and 250 read cycles for the reverse read.

### Sequencing data processing and microbiota analyses

The process of sequencing results generally follows the QIIME 2 (v2023.2) pipelines if not indicated elsewhere^67^. ASVs were generated using the DADA2 method^68^. Reads were rarified to 10,000 reads per sample before proceeding to further analysis. At the ASV level, alpha diversity indices and Bray-Curtis distance were calculated. The core ASV was defined as present in half of the samples in the subgroup. PERMANOVA and further manipulation and analysis of the distance matrix and principal coordinates were conducted using the Python package scikit-bio v0.5.9^69^.

### Taxon annotation and analysis

Taxon annotation was performed following the classify-consensus-vsearch pipeline based on VSEARCH against the SILVA database (v138)^70,71^. For analysis at the phylum level, phyla with mean abundance of less than 0.1% were assigned to minor phyla. For analysis at the ASV level, ASVs with a median abundance of zero both in saliva from control mice and in saliva from *H. parainfluenzae*-inoculated mice were assigned to minor ASVs, with the phylogenetic tree being constructed following the align-to-tree-mafft-iqtree pipeline. ANCOM-BC with FDR correction was conducted for analysis of differential abundance with statistical significance determined as *q* < 0.1^72^.

### CD8 T cell proliferation assay and supernatant cytokine determination

The A253 cells (ATCC HTB-41), derived from human submandibular glands with epithelial morphology and structure, were cultured in McCoy’s 5 A medium (ATCC) containing 10% fetal bovine serum (FBS)^33^. *H. parainfluenzae*, acquired from the National Taiwan University Hospital (Taipei, Taiwan), was heat-treated for 2 h at 56 °C to inhibit bacterial growth and resuspended in Dulbecco’s PBS^24^. A253 cells were conditioned with heat-inactivated *H. parainfluenzae* at a bacteria-to-cell ratio of 100:1 for 24 h.

CD8 T cells were isolated from the peripheral blood mononuclear cells from healthy donors by negative selection (BD Imag™ Human CD8 T Lymphocyte Enrichment Set). The isolated CD8 T cells were stained with 5 μM CFSE for 10 min and then washed twice with the T cell culture medium (RPMI-1640 with 1% L-glutamine, 1% penicillin–streptomycin, 10% FBS, 10 mM HEPES, and 50 μM β-mercaptoethanol). Following mitomycin treatment to preconditioned A253 cells, they were cocultured with CD8 T cells at a ratio of 1:5 with 1 μg/ml of anti-CD3/CD28 antibody (BD Biosciences) for 4 days. Cell proliferation was determined by flow cytometry. Supernatant IFN-γ and IL-4 were determined by a Cytometric Bead Array (BD Biosciences).

### Statistical analysis

The Student’s *t*-test was used to analyze the salivary abundance of *H. parainfluenzae* in human subjects, salivary flow rate in mice, and the frequency of IFN-γ^+^ or IL-4^+^ T cells. The Mann-Whitney U test was applied to focus score and area analyses. Correlation analyses between salivary flow rate and focus score or area were conducted using linear regression with Pearson’s correlation coefficient.

For alpha-diversity indices, the Mann-Whitney U test was used for non-paired analyses, while the Wilcoxon rank-sum test was applied for paired analyses within individual groups. Comparisons of paired changes between control and *H. parainfluenzae*-inoculated mice also used the Mann-Whitney U test.

For relative abundance analyses of selected targets (e.g., specific ASVs, phyla, phylum-to-phylum ratios, and species), the Mann-Whitney U test was applied to non-paired data, while the Wilcoxon rank-sum test was used for paired analyses within groups. Similarly, paired changes between groups were compared using the Mann-Whitney U test. CD8 T cell proliferation and supernatant cytokine levels were analyzed using the Student’s *t*-test.

Statistical analyses were performed using the Python packages statannotations (v0.6) and SciPy (v1.10.1). Normality was assessed using the Shapiro-Wilk test when appropriate and non-parametric tests were employed if normality was violated. Confidence interval estimates were generated using the Python package seaborn (v0.12.2) employing the bootstrap method^73^. Statistical significance was determined as *p* < 0.05.

## Supplementary information

Supplementary Information

## Data Availability

The 16S sequencing data have been deposited in the National Center for Biotechnology Information Sequence Read Archive under accession number PRJNA1096303. Supporting data for this study are available in the supplementary information.
